# Quality of life, burden of treatment, safety, and avoidance of future events (QBSAfe) protocol: a pilot study testing an intervention to shift the paradigm of diabetes care

**DOI:** 10.1186/s40814-021-00935-8

**Published:** 2021-11-08

**Authors:** Jennifer E. Clark, Kasey R. Boehmer, Maggie Breslin, Shanzay Haider, Weronika Pasciak, Derek Gravholt, Brianna B. Sanchez, Sandra A. Hartasanchez, Omar M. El Kawkgi, Victor Montori, Kasia J. Lipska

**Affiliations:** 1 Knowledge and Evaluation Research Unit, Mayo Clinic, Rochester, MN USA; 2 The Patient Revolution, Rochester, MN USA; 3 Section of Endocrinology, Yale University School of Medicine, PO Box 208020, New Haven, CT 06520 USA; 4 Frank H. Netter MD School of Medicine, Quinnipiac University, Hamden, CT USA

**Keywords:** Diabetes, Patient-centered, Conversation aid, Clinical toolkit, Chronic disease management

## Abstract

**Background:**

Diabetes care has been traditionally focused on targeting certain levels of glycemic control. This narrow emphasis may impose burdens on patients, including high treatment costs, illness-related work, or side effects from medications, while leaving other patient needs and goals under-addressed. The authors aim to shift the paradigm of care for people with diabetes, to focus on quality of life, burden of treatment, safety, and avoidance of future events: the QBSAfe domains.

**Methods:**

We describe a single-arm pilot study to assess the feasibility and acceptability of using the QBSAfe agenda setting kit (ASK) during routine clinical visits. The set of 14 conversation aid cards was co-developed with patients, family caregivers, and clinicians. The ASK will be used in the context of a clinic visit, which will be recorded by members of the study team to identify patterns of clinician-patient conversations. Feasibility will be measured by the number of participants recruited, time to goal accrual, and completeness of data collection; acceptability will be assessed using post-visit surveys of patients and clinicians. A subgroup of patients will be invited to participate in post-visit qualitative semi-structured interviews for additional feedback. This study will be conducted across three medical centers in the Midwest and East Coast of the USA.

**Discussion:**

Current healthcare infrastructure and associated demands and pressures on clinicians make changes in care difficult. However, this intervention has the potential to shift conversations during clinical encounters so they can address and directly respond to patient needs, symptoms, and capacity. As part of the QBSAfe ASK, the authors are also actively collaborating with a variety of stakeholders to create tools to help clinicians respond more effectively to patient concerns as they are raised during the clinical encounters. Additional insights about the use of the QBSAfe approach in the virtual space will be gathered during the process of our study due to restrictions imposed upon face to face visit during the COVID-19 pandemic.

**Trial registration:**

ClinicalTrials.gov, NCT04514523. Registered 17 August 2020—retrospectively registered.

## Background

Nearly 30 million adults in the USA have been diagnosed with diabetes mellitus, and most take medications to control their hyperglycemia [1, 2]. People with type 2 diabetes tend to be older in age and are frequently affected by multiple other comorbidities or functional impairments, and one or more geriatric conditions. As a result, glycemic management in this population can be inherently challenging. Multiple chronic conditions and impairments may create barriers to self-management of complex tasks (such as insulin administration and dosing) and may leave patients overwhelmed and overburdened by the work required to manage their conditions. More intensive management may also increase risk of adverse events such as hypoglycemia, which may beget further disease-related distress and burden [3, 4]. Social isolation, limited social support, poor financial, physical and mental health, and personal and social complexities limit the capacity of patients and caregivers to shoulder the mushrooming treatment workload [5].

Prior studies demonstrated that patients with diabetes experience high treatment burden and routinely discuss this with their clinicians, but these discussions rarely lead to any efforts to address this burden [6]. It is also known that hospitalizations for hypoglycemia pose a significant health threat for older adults and are now more common than admissions for hyperglycemia [7]. These findings suggest that a paradigm shift is needed from the narrow focus on reaching hemoglobin A1c targets to a more holistic approach that responds to patient needs, fits within the patient’s context and capacity, and prevents iatrogenic harms.

In an effort to shift the paradigm of care for people with diabetes to a more patient goal-centric approach, we developed and propose to test the QBSAfe agenda setting kit (ASK). The QBSAfe ASK was developed to help clinicians care for people with diabetes and focus on what is important to them. The domains of QBSAfe include quality of life, burden of treatment (medication administration, costs, and monitoring), safety, and avoidance of future events (including hypoglycemia and other adverse events, as well as diabetic complications). Using this approach, we aim to turn clinical encounters into sessions during which clinicians and patients work together to co-create plans that respond to problems identified during each visit. We hypothesize that the use of QBSAfe will allow clinicians to hear the story from the patient’s point of view and identify problems/issues that need to be addressed, so that the patient and clinician may prioritize together what issues demand action.

## Methods

The goal of the QBSAfe ASK intervention is to help clinicians elicit and respond to patient needs in diabetes care. The aim of our study is to test this intervention in routine ambulatory clinical settings, and determine its ability to achieve its goal in addition to evaluating the acceptability and feasibility of its use in clinical care. This is a mixed-methods single-arm pilot study including survey data, demographic data, intervention use metrics, and qualitative interviews to determine the impact of the intervention.

### Development of the intervention

The first iteration of the intervention involved use of brief surveys about overall quality of life, diabetes-related quality of life, difficulties with management of diabetes, hypoglycemia, and social support—all of which have been identified both from the literature and clinical experience as aspects of life that people with diabetes deemed to be important [8–13]. In early stages of our work, we found that patients were able to complete the surveys fairly easily, but this did not lead to discussions in which the issues highlighted on the surveys were brought up or discussed during the conversation. We hypothesized that the volume of questions and the format were not conducive to conversation or problem-solving.

In response to these early results and with the intent of bringing the contents of the surveys into a conversation during the clinical encounter (where they could be turned into action), we turned our attention toward conversation cards, which can be used to engage patients in agenda setting and care planning [14–18]. We developed 12 cards that corresponded to the most important topics to patients living with diabetes reflected by the surveys we collected. These card prototypes were then refined and field-tested in conversations with patients with diabetes. We also obtained input from a patient advisory group. As a result, we added 2 additional cards, about successes in diabetes care, based on feedback from and observations of their use by diabetes nurse educators.

### Description of the intervention

**Fig. 1:**
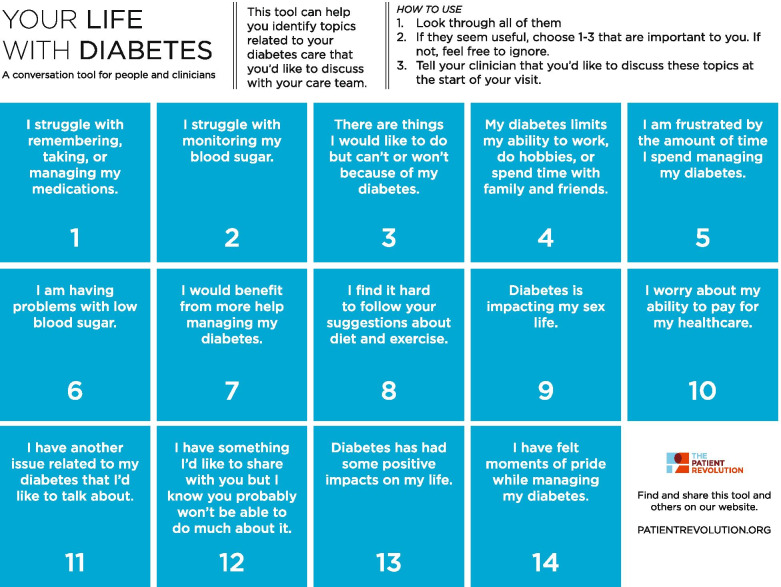
QBSAfe agenda setting kit conversation cards

The intervention consisted of 14 conversation cards: QBSAfe agenda setting kit (ASK). The cards included in this initial prototype kit address aspects of each of the QBSAfe domains (quality of life, burden of treatment, safety, and avoidance of future events). These include “I struggle with remembering, taking, or managing my medications,” and “I worry about the ability to pay for my healthcare” (Fig. 1). These cards can be printed as sets of 14 discrete reusable cards or as a single disposable sheet with the 14 cards, so that they can be handed to the patient for review prior to the start of their scheduled clinic visit. The digital version of the cards is available online on the patient revolution website (url: https://patientrevolution.org/qbsafe). In addition to displaying each of the 14 cards, the website also includes audio playback of each card’s text and a video demonstration of their use in a mock clinical encounter. The ASK may be presented to the patient as a set of cards just prior to the onset of the visit (e.g., by the medical assistant or by the study team member), or shared by the patient’s clinician at the time of the clinical encounter. Alternatively, during virtual visits, the set of cards can be accessed by the patient via a textable link ahead of the clinic visit. Although this prototype may undergo additional iterations in the future, none are planned over the course of this study.

### Participants

The study’s target accrual is 100 adult patients with diabetes mellitus (type 1 or type 2); recruitment is ongoing at the time of this writing. Included patients are eligible if they (1) are > 18 years of age, (2) have a diagnosis of diabetes mellitus, and (3) are scheduled for an appointment with a clinician participating in the study. Patients are excluded if they (1) do not speak English, (2) have a severe vision or hearing impairment, or (3) if they are unable to give informed consent for any reason. Enrolled clinicians may also choose to exclude a patient from the study based on their judgment of patient suitability for the study. Clinicians are eligible if they are a practicing physician, advanced practice practitioner, or diabetes nurse educator who regularly cares for patients with diabetes, including those specializing in the fields of primary care/family medicine, geriatrics, internal medicine, and endocrinology. The study team reached out to clinicians who were known to regularly see patients with diabetes, to assess interest in participating.

All patients and clinicians who participate in the study will be asked for their permission to use the data collected through their participation in the study (such as audio or video recordings) for ongoing registry purposes for future research (which would also be subject to additional institutional review board approval), training, and educational purposes. Basic demographic data, including age, sex, race, and insulin use status, will also be obtained from the patient participants’ electronic medical records at the time of enrollment.

### Recruitment procedures and observations of clinical encounters

This pilot study will take place at three sites: one academic medical center in the Midwest of the USA, and two on the East Coast of the USA (a large academic center and a smaller non-profit health system). Potential patient participants will be screened from the schedules of enrolled clinicians. Upon obtaining approval by the clinician, the potential participants are to be contacted by a member of the study team prior to their scheduled appointment time via phone and/or email correspondence. If the patient agrees to participate and provides electronic or paper consent, the scheduled clinical encounter will be recorded. The recording is conducted using a portable GoPro camera, operated by the clinician or by a study team member. It will be explicitly noted to each party at the time of the visit when a recording device will be used; the clinician and patient will have the ability to discontinue the recording at any time during the visit if either party wishes.

**Fig. 2:**
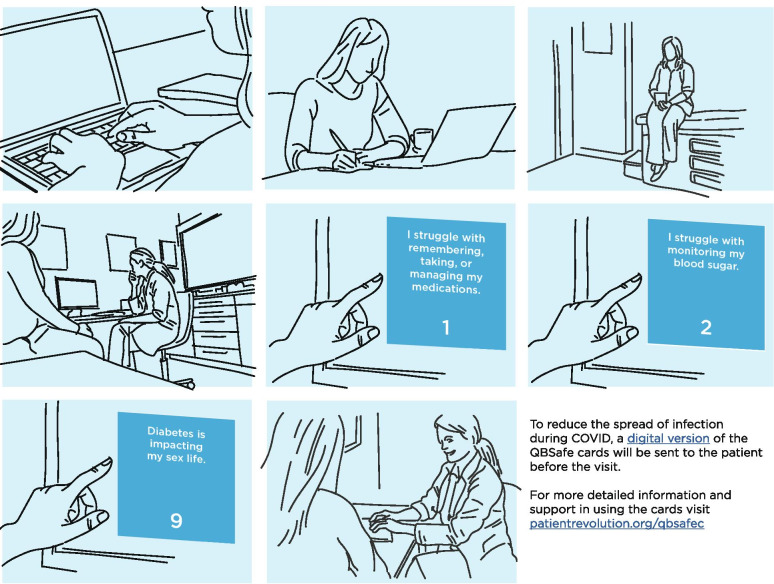
Storyboard depicting patient reviewing digital QBSAfe conversation cards prior to their scheduled visit, and discussing with their clinician

Patients will be asked to review the QBSAfe cards via electronic format before their scheduled visit, or via paper format at the time of their scheduled visit (prior to their clinician entering the exam room). After reviewing the topics presented on the cards, patients will be asked to choose 0‑3 cards to discuss with their clinician that seemed relevant to their life with diabetes at that time. If the patient does not bring up the topics on the cards at the time of the visit, the clinician will be instructed to ask about which cards they chose. Other than this prompt, the clinician is encouraged to address the topics on the cards as they best see fit at the time of the clinical encounter (Fig. 2).

Recordings of clinical encounters will be used to further review and debrief with the team members on the protocol that could not be present at the time of the encounter. The recorded conversations will also be used to perform analysis of the communication patterns and themes identified during the visit. Using these thematic analyses, the study team will explore the impact of the QBSAfe ASK on clinician-patient conversations and obtain further information about the acceptability/feasibility of the intervention. These videos, in combination with input of study team members/other stakeholders, and experiences to date, will also assist with possible adjustments to the toolkit as deemed appropriate to better facilitate patient-centered discussion.

### Protocol modifications due to COVID-19 pandemic

Changes in healthcare access and delivery at the onset of the COVID-19 pandemic necessitated changes to our study protocol. The study teams transitioned to a primarily remote recruiting strategy, using remote consenting procedures (electronic consenting documents) as much as possible to better facilitate social distancing precautions. Although the QBSAfe cards were initially intended to be used as 14 discrete cards, they were transformed into a digital format accessible via the internet (https://patientrevolution.org/qbsafe). Enrolled patient participants are instructed to review these digital “cards” before their scheduled clinic visit, and to take note of which topics they wanted to discuss with their clinician. The protocol is flexible to account for changes in COVID-19 infection rates at each site and safety of in-person visits.

Recording procedures also changed as a result of the pandemic. Recording of clinical encounters at the Midwest site was initially performed via secure teleconferencing with the use of the webcams in the exam rooms (completely remote recording strategy), followed by transition to the use of cameras (GoPro Hero 7) in the examination room set up prior to the clinic visit time by a team member on site. Planned enrollment numbers did not change as a result of the pandemic.

### Evaluating feasibility and acceptability

As part of our pilot study of the QBSAfe ASK, we plan to assess the feasibility and preliminary acceptability/effectiveness of the intervention in clinical practice. To evaluate feasibility, we will measure willingness to participate in the study by (1) percent of participants (both patients and clinicians) who consent to enroll of all those who were approached and eligible; (2) percentage of participants who completed the study procedures, used QBSAfe toolkit (i.e., if cards were chosen, and which cards), and completed the short survey after completion of the clinic visit; and (3) time to recruit 100 subjects across 3 sites. Acceptability will be measured using survey data, including three acceptability questions for both patient and clinician participants, administered after each observed or recorded clinical encounter (Table 1). Percentage of patients and clinicians who find the intervention acceptable (strongly agree/agree responses) will be used to determine intervention acceptability in this pilot study. Fidelity will be assessed by video analysis.

**Table 1:** Acceptability questions

**Clinician survey** (answer with Strongly Agree/Agree/Neutral/Disagree/Strongly Disagree)	
I felt confident in responding to issues raised by the patient using the conversation cards.	
I would like to use these cards with a patient like this next time.	
I would recommend these cards to my colleagues when caring for patients similar to this one.	
**Patient survey** (answer with Strongly Agree/Agree/Neutral/Disagree/Strongly Disagree)	
These cards helped me talk about my situation during my visit.	
I would like to have these cards available to me at my next visit.	
I believe other patients like me would benefit from these cards.	

For additional data regarding the acceptability, effectiveness, and feasibility of the conversation cards, the study team also plans to perform at least 30 qualitative semi-structured interviews with a purposive sample of study patients. The purposive sampling strategy will be informed by patient age, gender, and whether the patient is taking insulin or not. During the interviews, patients are asked to first briefly describe their experience living with diabetes, then describe the clinic visit experience and the use of the conversation toolkit, including what went well, what could have gone better, and how the use of the toolkit impacted the clinic visit and clinician recommendations. These qualitative interviews will be audio recorded and transcribed. Outcomes are summarized in Table 2. Data regarding patient demographics, including age, sex, and insulin use, will also be collected for this study.

**Table 2:** Main outcomes

**Acceptability**	
Patient and clinician responses to post-visit survey questionnaires	
**Feasibility**	
Percentage of approached and eligible patients and clinicians who consent to enroll in the study	
Percentage of participants who completed the study procedures (visit recorded, post-visit surveys completed)	
Percentage of recorded visits in which the QBSAfe toolkit was used	
Time to recruit 100 subjects across 3 sites	
**Qualitative analysis**	
Identification of themes and further assessment of feasibility and acceptability of QBSAfe toolkit through post-visit interviews of at least 30 patients	

### Analysis

Transcripts of the clinical encounters and post-visit interviews will be analyzed using both inductive and deductive content analysis [19–21]. Deductive codes will include noting the cards used and issues expected to be brought up by the cards. Remaining codes will be inductively derived from the transcribed data. The coding team will be made up of at least three coders. Coders will first code one transcript independently and meet together to discuss developing codes that will be noted in a standardized codebook. This process will continue for three to six transcripts until it is determined that coders are sufficiently calibrated in their coding and no new codes are emerging from the data. At that time, coding will be continued independently by one coder per transcript, with periodic team check-ins to discuss progress and resolve any further questions related to coding via team consensus. After coding is completed, the team will summarize themes that have emerged from the data. These themes will then be used to develop a coding scheme to deductively code all video-recorded encounters collected in the study. This video analysis is intended to quantify the impact of the QBSAfe toolkit on diabetes conversations and care. The analysis process will be overseen by an investigator experienced in qualitative and videographic analysis methods.

Before progressing to a larger trial, study team will review the data on acceptability and feasibility to assess the following set of progression criteria: recruitment (> 50%), form completion (> 80%), and acceptability (> 80%). If below target, study team will work to revise implementation of the trial procedures to improve and optimize these feasibility and acceptability endpoints.

## Discussion

Diabetes care has traditionally focused on targeting and maintaining specific levels of glycemic control (average glucose or HbA1c) to reduce the risk of diabetic complications. However, this approach can be problematic. Therapies to achieve target HbA1c levels may impose significant burdens on patients (taking medications, injecting insulin, monitoring blood sugars, adjusting diet and exercise), may create or add to financial problems (increase premiums or out-of-pocket costs), may lead to hypoglycemia or other adverse events, and may not necessarily improve quality of life [20, 21]. Older patients are especially vulnerable to the added burdens of diabetes care, as they often already have multiple other comorbidities, symptom burden, and/or functional impairments impacting quality of life [22, 23]. Therefore, implementation of evidence- and guideline-based diabetes treatments without attention to these additional factors or patient-reported outcomes may increase distress and could worsen how the patient feels or functions in their day-to-day life.

The balance of time and effort needed to manage a chronic disease such as diabetes and the ability to perform this work can be conceptualized in the context of the cumulative complexity model [5]. The capacity to do “patient work” is drawn from the same capacity to do the work of everyday life (e.g., work, hobbies). When the workload of one’s diabetes care exceeds the capacity to enact it, it becomes burdensome and causes distress. Overwhelmed patients may then reduce their adherence to treatment and worsen their hyperglycemia, leading to a vicious cycle of intensification of treatment and therefore disease-related work (e.g., new medications, more complex regimens, increased monitoring) with rising HbA1c. At the same time, other symptoms (from diabetes or associated comorbidities) remain unaddressed, further reducing the patient capacity to respond. The end result is a greater burden of treatment, reduced capacity, and greater diabetes distress.

With our group’s work thus far, and with the data from this study, we hypothesize that a shift to an approach focused on patient context and goals will turn clinical encounters into problem-solving sessions with solutions co-developed between the patient and their clinician. We seek to improve diabetes care among individuals experiencing diabetes distress by drawing clinicians’ attention to the extent to which diabetes and its treatment affect their patient’s symptoms and overall well-being (QBSAfe approach). This will ideally lead to better treatment fit, adherence, and patient-centered outcomes.

To accomplish these goals, our protocol describes a single-arm pilot study to assess the feasibility and acceptability of a new conversation tool, the QBSAfe ASK. Conversation cards, although seemingly simple and low-tech, can be an effective way to engage patients in care and to tackle difficult issues. They have been used to that effect in the management of obesity among adolescents and in end-of-life decision-making [14–17]. Strengths of our study include the collaborative- and evidence-based development of our intervention, as well as the mixed-methods research design, including collection of quantitative data on feasibility and acceptability of the intervention, and qualitative analysis of clinical encounters and post-visit interviews to understand the impact of QBSAfe on patient experience of care. However, we foresee challenges in the recruitment and consenting process related to the COVID-19 pandemic and need for social distancing procedures. Preliminary experience thus far suggests that patient interaction with the digital “cards” versus the physical set of QBSAfe cards may lead to different responses or reactions from the patient participants. The environment of the virtual encounter may also affect the collaborative nature of the patient-clinician conversation, which warrants future study.

Despite these challenges, we expect that the results of our study will not only inform helpful strategies to improve patient-clinician conversations, but also provide additional insights about the potential differences in engagement and conversation between the virtual and physical clinical encounter spaces. These findings will help to inform future iterations of our conversation aid toolkit with the ultimate goal of testing its use versus usual care in a randomized trial.

## Data Availability

Data from this study that can be fully de-identified are available from the corresponding author on reasonable request.

## Abbreviations

QBSAfe: Quality of life, burden of treatment, safety, and avoidance of future events
ASK: Agenda setting kit
